# Role of pancreatic stellate cells in chemoresistance in pancreatic cancer

**DOI:** 10.3389/fphys.2014.00141

**Published:** 2014-04-09

**Authors:** Joshua A. McCarroll, Stephanie Naim, George Sharbeen, Nelson Russia, Julia Lee, Maria Kavallaris, David Goldstein, Phoebe A. Phillips

**Affiliations:** ^1^Tumour Biology and Targeting Program, Lowy Cancer Research Centre, Children's Cancer Institute Australia, University of New South WalesSydney, NSW, Australia; ^2^Australian Centre for Nanomedicine, University of New South WalesSydney, NSW, Australia; ^3^Pancreatic Cancer Translational Research Group, Lowy Cancer Research Centre, Prince of Wales Clinical School, University of New South WalesSydney, NSW, Australia

**Keywords:** pancreatic cancer, chemoresistance, pancreatic stellate cells, stroma, fibrosis, hypoxia

## Abstract

Pancreatic cancer is highly chemoresistant. A major contributing factor is the characteristic extensive stromal or fibrotic reaction, which comprises up to 90% of the tumor volume. Over the last decade there has been intensive research into the role of the pro-fibrogenic pancreatic stellate cells (PSCs) and their interaction with pancreatic cancer cells. As a result of the significant alterations in the tumor microenvironment following activation of PSCs, tumor progression, and chemoresistance is enhanced. This review will discuss how PSCs contribute to chemoresistance in pancreatic cancer.

## Introduction

Pancreatic cancer is a highly aggressive malignancy with a notoriously dismal prognosis. Contributing to ~227,000 annual deaths worldwide, this insidious disease is the fourth leading cause of cancer-related death in developed countries (Raimondi et al., 2009; Hidalgo, 2010; Vincent et al., 2011). Remarkably, 80–85% of patients present with unresectable and incurable tumors, putting the median survival period at <6 months and the overall 5-years survival rate at <5% (Hidalgo, 2010; Vincent et al., 2011).

A major contributor to this poor clinical outcome is pancreatic cancer's prominent chemoresistance (Zalatnai and Molnar, 2007; Wang et al., 2011). In fact, the best treatments only prolong life by ~8–16 weeks (Wolfgang et al., 2013). Until recently, most research efforts focused solely on cancer cells. However, significant stakeholders in this chemoresistance are key pro-fibrogenic cells of the pancreas known as pancreatic stellate cells (PSCs), which when co-opted and activated by cancer cells, orchestrate the strong desmoplasia that characterizes pancreatic cancer (Apte et al., 2004; Bachem et al., 2005). The resultant stromal landscaping yields an exclusive microenvironment where cross-talk between cancer-associated human PSCs (CA-hPSCs) and cancer cells promotes local tumor progression, metastasis and chemoresistance (Apte et al., 2004; Bachem et al., 2005; Hwang et al., 2008; Vonlaufen et al., 2008a,b; Xu et al., 2010; Erkan et al., 2012; Phillips, 2012).

CA-hPSCs establish fibrosis via excessive extracellular matrix (ECM) deposition, which compresses and distorts intratumoural vasculature, causing hypoxia (Olive et al., 2009; Phillips, 2012; Stylianopoulos et al., 2012; Jacobetz et al., 2013). Hypoxia stimulates the epithelial-mesenchymal transition (EMT) of cancer cells, which is a more chemoresistant phenotype (Arumugam et al., 2009; Kikuta et al., 2010; Wang et al., 2011). Furthermore, the fibrosis sequesters chemotherapeutics in the stromal compartment, impairing successful drug delivery to cancer cells (Olive et al., 2009; Provenzano et al., 2012; Jacobetz et al., 2013). Despite being in a hostile microenvironment replete with cytotoxic drugs and hypoxia, CA-hPSCs not only survive, but actually thrive and proliferate causing the tumor microenvironment to occupy up to 90% of the tumor volume (Li et al., 2010; Neesse et al., 2011; Michl and Gress, 2013).

A major limitation to our understanding of the role of CA-hPSCs in chemoresistance is their survival mechanisms in this noxious microenvironment. However, mounting evidence suggests CA-hPSCs are both direct and indirect drivers of pancreatic cancer chemoresistance and spread, and thus their inhibition may potentiate current chemotherapy and intercept tumor-facilitatory bidirectional interactions.

## A growth permissive microenvironment

The histopathological hallmark of pancreatic cancer that underlies its aggressiveness is the severe desmoplastic and fibroinflammatory reaction which generates a high stromal-to-epithelial ratio (Li et al., 2010). In fact, pancreatic ductal adenocarcinoma (PDAC), which comprises >85% of all pancreatic cancer subtypes, is one of the most stroma-rich malignancies (Bardeesy and Depinho, 2002; Feig et al., 2012). Despite PDAC demonstrating similar chemosensitivity as other cancers *in vitro*, PDAC patients are less responsive to chemotherapeutics than other cancers (Li et al., 2010), implicating the unique microenvironment in PDAC's chemoresistance. Such desmoplasia facilitates a mechanopathology known as growth-induced solid stress (GISS), resulting in collapsed or compressed intratumoural blood vessels or lymphatics, which respectively lead to increased hypoxia and interstitial fluid pressure (IFP); both attenuate chemosensitivity (Stylianopoulos et al., 2012). Non-invasive quantification of physiological parameters in human pancreatic tumors had confirmed that blood flow is reduced and metabolic activity is increased relative to normal pancreas (Komar et al., 2009). This recapitulates the significant consequences of GISS, as deficient vasculature reduces blood flow, yielding hypoxia (Stylianopoulos et al., 2012). Subsequently, stromal and cancer cells undergo aerobic glycolysis—“The Warburg Effect”—leading to their increased metabolic activity, as a means of surviving in this hypoxic microenvironment (Tod et al., 2013).

**Figure 1:**
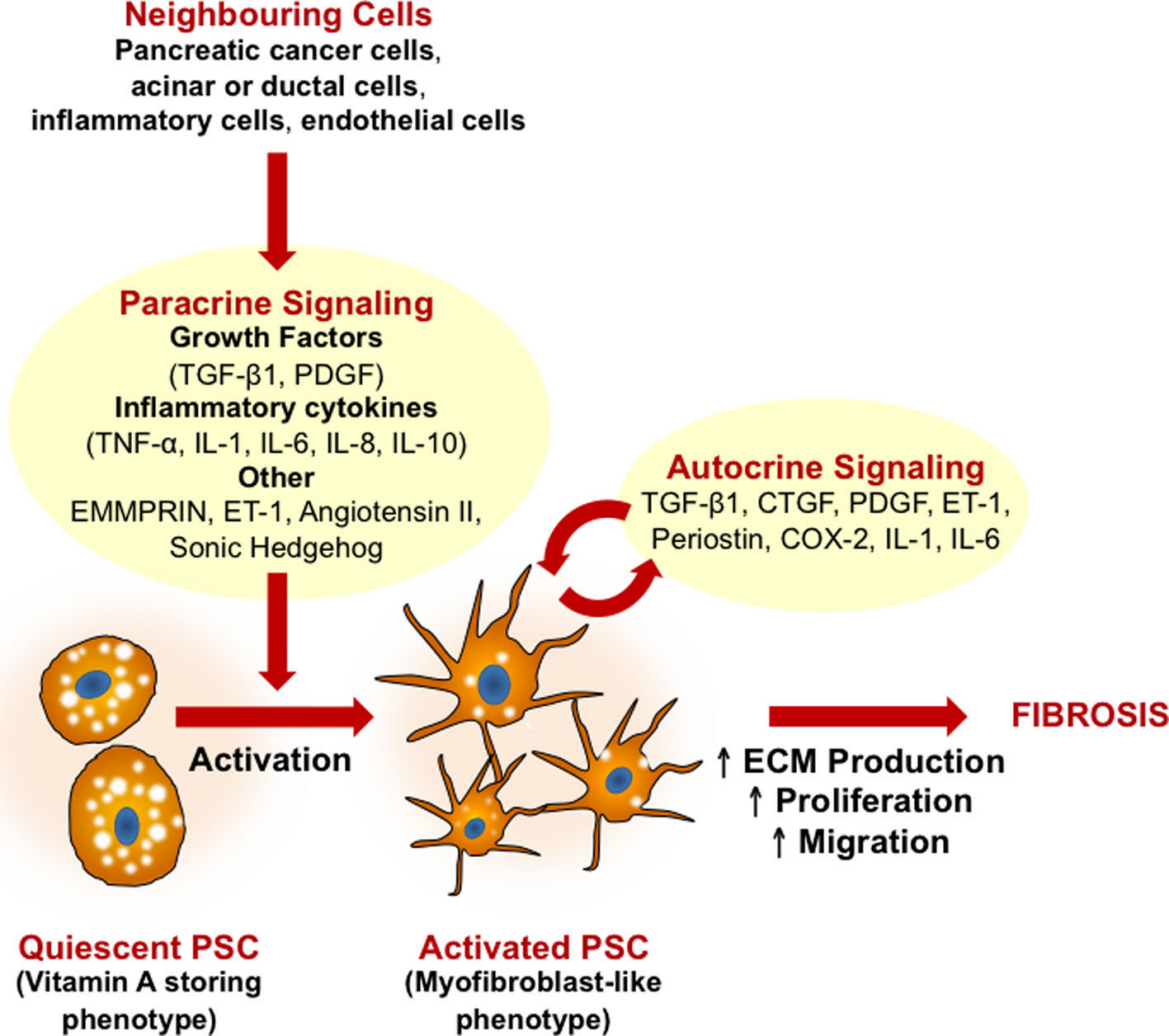
**Mechanisms of pancreatic stellate cell (PSC) activation.** A central feature of PSCs is the transformation from a quiescent (fat storing phenotype) to an activated (myofibroblast-like phenotype) state. Growth factors and pro-inflammatory cytokines released by neighboring cells (pancreatic cancer cells, injured acinar or ductal cells, inflammatory cells, and endothelial cells) all induce PSC activation. Activated PSCs can then perpetuate this activation state via autocrine stimuli, leading to increased proliferation, migration, and excessive ECM production. In pancreatic cancer, activation of PSCs leads to the production of extensive fibrosis which in turn contributes to disease progression, metastases and chemoresistance. CTGF, connective tissue growth factor; COX-2, cyclooxygenase-2; ECM, extracellular matrix; EMMPRIN, extracellular matrix metalloproteinase inducer; ET-1, endothelin 1; IL, interleukin; PDGF, platelet derived growth factor; TGF-β, transforming growth factor β; TNFα, tumor necrosis factor α; TRAIL, TNF-related apoptosis-inducing ligand.

The stroma in PDAC is composed of abundant ECM proteins, such as collagen and hyaluronan, along with nerves, blood, and lymphatics, and a versatile cellular population including inflammatory cells and activated PSCs (Erkan et al., 2010; Neesse et al., 2011). Several paracrine and autocrine factors induce stroma production (Li et al., 2010; Neesse et al., 2011; Phillips, 2012) (Figure 1).

## Pancreatic stellate cells—key fibrogenic cells in pancreatic cancer

In health, PSCs are quiescent, represent ~4% of the cell population, express desmin, and glial fibrillary acidic protein and store cytoplasmic vitamin A-containing lipid droplets (Apte et al., 1998, 2006). The latter markers are PSC-specific, distinguishing them from normal pancreatic fibroblasts. Disease onset heralds the accumulation of reactive oxygen species, cytokines and growth factors secreted by injured cells, which can all activate PSCs (Apte et al., 1999; Mews et al., 2002; Gao and Brigstock, 2005; Kordes et al., 2005; Vonlaufen et al., 2010) (Figure 1).

Such paracrine stimuli induce quiescent PSCs to transdifferentiate into a myofibroblast-like phenotype, gaining expression of α-smooth muscle actin (α-SMA) and losing the anti-fibrogenic lipid droplets (Apte et al., 1998). These morphological changes are accompanied by functional changes (Figure 1), such as: (1) increased proliferation and migration (Apte et al., 1999; Schneider et al., 2001; Mews et al., 2002; Phillips et al., 2003b; Omary et al., 2007); (2) excessive synthesis of ECM proteins as well as matrix metalloproteinases (MMPs) and their inhibitors (Apte et al., 1999, 2004; Schneider et al., 2001; Phillips et al., 2003a; Bachem et al., 2005); and (3) secretion of growth factors and cytokines involved in autocrine loops that perpetuate PSC activation (Shek et al., 2002; Ohnishi et al., 2003; Aoki et al., 2006a,b; Omary et al., 2007; Jiang et al., 2009).

## Bidirectional interactions between pancreatic stellate cells and cancer cells

Substantial evidence corroborates the notion that bidirectional interplay occurs between cancer cells and CA-hPSCs, commensally facilitating tumor progression (Apte et al., 2004; Bachem et al., 2005; Hwang et al., 2008; Vonlaufen et al., 2008a; Xu et al., 2010). Cancer cells recruit PSCs via mitogenic and fibrogenic factors which promote PSC activation, proliferation, migration and ECM remodeling capability. For instance, supernatants from PDAC cell lines stimulated PSC proliferation and ECM synthesis in a dose-dependent manner, with these effects abrogated by neutralizing antibodies against platelet-derived growth factor (PDGF), fibroblast growth factor, and transforming growth factor-β1 (TGF-β1) (Bachem et al., 2005). Also, PSC synthesis of MMP-2, a protein crucial for basement membrane degradation, is increased by ECM metalloproteinase inducer (EMMPRIN), which is secreted by cancer cells (Schneiderhan et al., 2007). Such modulation of the ECM is critical for degradation of basement membrane and may influence cancer progression.

PSC recruitment is consequential for cancer cell behavior, as stellate or cancer cell-derived growth factors, cytokines and ECM components are sequestered in a fortified niche. For example, Bachem et al. (2005) originally demonstrated that subcutaneous co-injection of PSCs and cancer cells into nude mice accelerated tumor growth relative to solely injecting cancer cells. This was substantiated in an orthotopic mouse model which employed intra-pancreatic co-injections of PSCs and cancer cells, replicating the aforementioned augmented tumor growth and showing enhanced local and distant metastases relative to solely injecting cancer cells (Vonlaufen et al., 2008a). Xu et al. (2010) also demonstrated that CA-hPSCs co-migrate with metastasizing cancer cells, which is likely to aid cancer cell seeding and growth. Furthermore, PSC-conditioned medium increased proliferation, migration, invasion, and chemoresistance of cancer cells and reduced their apoptosis *in vitro* (Hwang et al., 2008; Vonlaufen et al., 2008a; Gao et al., 2010), while orthotopic co-injection of PSCs and cancer cells increased primary tumor incidence and size *in vivo* (Hwang et al., 2008). Moreover, co-culturing with PSCs promoted EMT of PDAC cells (Kikuta et al., 2010). Evidence by Watanabe et al. (2003) also demonstrated that extensive intratumoural fibroblastic cell proliferation correlates with a poorer disease outcome in pancreatic cancer patients. This significant evidence suggests that abolishing CA-hPSCs or their activity may reduce PDAC's aggressiveness, necessitating an improved understanding of CA-hPSC survival mechanisms.

## Pancreatic stellate cells and chemoresistance of pancreatic cancer

**Figure 2:**
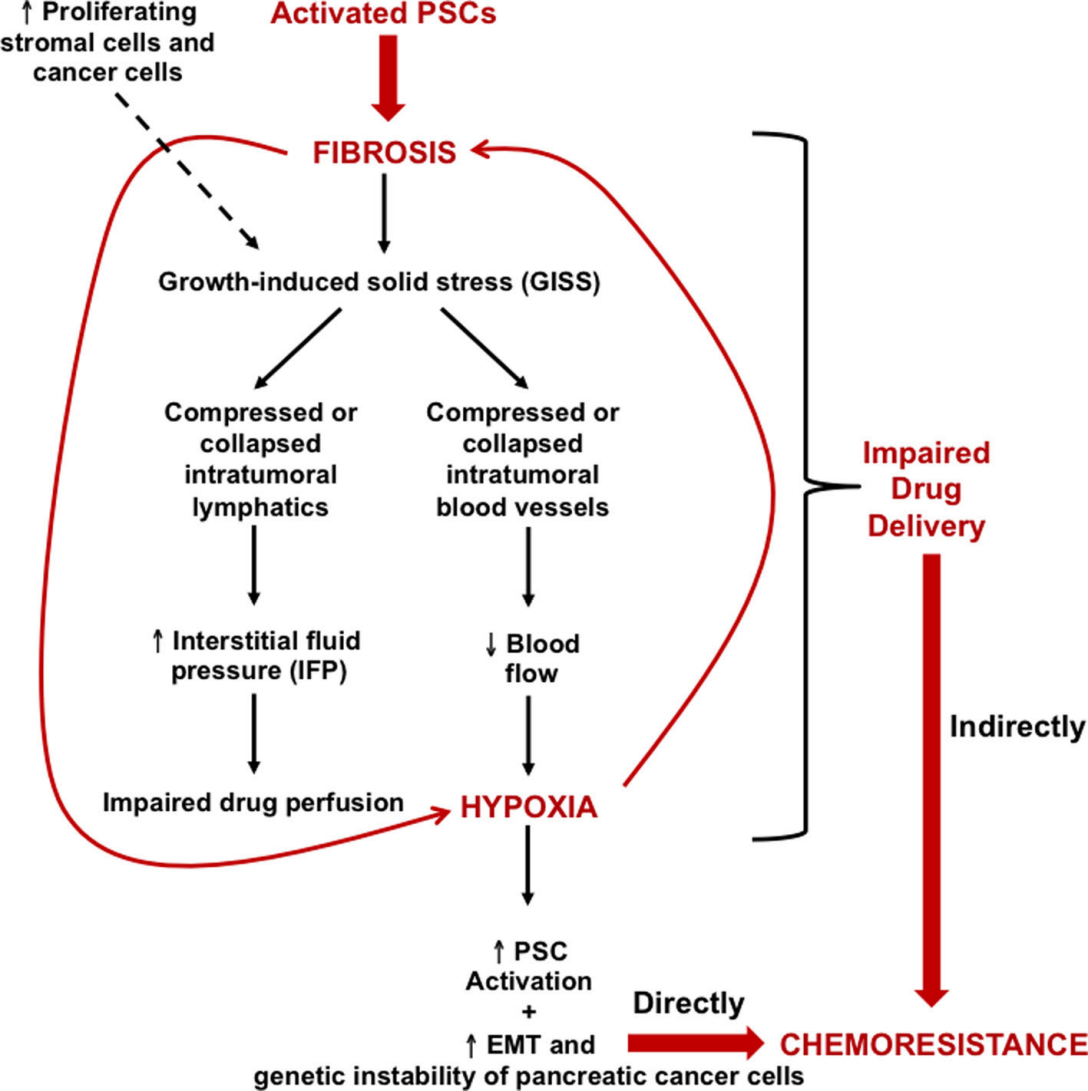
**Growth-induced solid stress (GISS), the hypoxia-fibrosis cycle and their contribution to chemoresistance.** Continuous PSC activation results in excessive ECM deposition, particularly of tensile-resistant fibrillar collagen as well as compression-resistant hyaluronan. This eventually leads to prominent fibrosis which, along with deformation caused by the number of proliferating stromal cells and cancer cells, results in GISS. Consequently, this reduces the caliber of intratumoral lymphatics and blood vessels. The former leads to increased IFP, which can impair drug perfusion, while the latter reduces blood flow, consequently leading to intrastromal and intratumoral hypoxia. This loops back onto PSCs, driving their activation and hence generating more fibrosis, which creates a hypoxia–fibrosis cycle. The cycle indirectly contributes to chemoresistance by impairing drug delivery to cancer cells. Moreover, hypoxia is capable of increasing the genetic instability and EMT of pancreatic cancer cells, directly fuelling their chemoresistance. EMT, epithelial-mesenchymal transition.

PDAC is highly refractory to chemotherapeutics (Wolfgang et al., 2013). While the cause of chemoresistance is multifactorial, three major processes have been distinguished: (1) reduced drug uptake; (2) increased energy-dependent drug efflux; and (3) alterations in cellular capabilities affecting drug cytotoxicity, such as reduced apoptosis and dysregulated drug metabolism (Szakacs et al., 2006; Zalatnai and Molnar, 2007). However, another major determinant of pancreatic cancer chemoresistance is the extensive fibrosis produced by PSCs, which results in significant intratumoural hypoxia and a self-perpetuating hypoxia-fibrosis cycle (Figure 2) (Koong et al., 2000; Evans and Koch, 2003; Erkan et al., 2009). CA-hPSCs extend this chemoresistant profile via the hypoxia-fibrosis cycle (Masamune et al., 2008; Phillips, 2012). This impairs drug delivery to cancer cells and stimulates their EMT and genetic instability, yielding a more chemoresistant phenotype (Arumugam et al., 2009; Kikuta et al., 2010; Wang et al., 2011).

Seminal proof-of-principle studies have substantiated the crucial influence of the fibrotic hypovascular stroma on GISS and chemoresistance in PDAC using genetically engineered mouse models. One study interrupted hedgehog signaling in the stroma (Olive et al., 2009), while two others enzymatically ablated hyaluronan, the main ECM determinant of the fibrotic stroma (Provenzano et al., 2012; Jacobetz et al., 2013). In all studies, the stroma was dramatically depleted leading to increased intratumoral vascular density and normalization of IFP. Following stromal depletion, administering chemotherapeutics such as gemcitabine or doxorubicin resulted in enhanced intratumoral drug perfusion, rendering tumors vulnerable to cytotoxicity, and hence inhibiting tumor growth and prolonging overall survival (Olive et al., 2009; Provenzano et al., 2012; Jacobetz et al., 2013). However, a Phase II clinical trial following the Olive et al. (2009) study ceased due to increased mortality in the treatment arm. This may have resulted from removal of the tumor-containing fibrotic barrier, encouraging the escape of aggressive clones which underwent metastatic evolution in response to the microenvironment's high selection pressure, however this needs further investigation. Regardless, these landmark studies elegantly showed that tumor-associated stromal collapse in mice is therapeutically effective. Hence, what if we deplete the stroma-producing CA-hPSCs rather than the stroma itself? Since stromal and cancer cells have been shown to contribute to GISS, their depletion can relieve it (Stylianopoulos et al., 2012).

In addition to the hypoxia-induced chemoresistance, PSCs can directly impact the response of cancer cells to chemotherapy. hPSC secretions have been shown to confer a chemoresistant cancer cell phenotype by (i) suppressing H_2_O_2_-induced apoptosis and increased survival of pancreatic cancer cells (Vonlaufen et al., 2008a); and (ii) decreased pancreatic cancer cell sensitivity to gemcitabine and radiation therapy (Hwang et al., 2008). These results were supported by Muerkoster et al. (2004), who demonstrated that pancreatic cancer cells co-cultured with PSCs are less sensitive to etoposide. In addition, pancreatic cancer cells cultured with ECM proteins produced by PSCs promoted resistance to 5-fluorouracil (5-FU), cisplatin, and doxorubicin (Miyamoto et al., 2004). However, a limitation in the field is that no studies have examined the influence of PSCs on proteins which protect tumor cells against chemotherapy agents (for example multi-drug resistant drug transporters).

It is important to note that in addition to the impact which stellate cells have on pancreatic cancer chemoresistance, it is now well established that the immune cells also impact on tumor progression and chemoresistance (reviewed in Evans and Costello, 2012; Hamada et al., 2013). Immune cells within the tumor microenvironment also activate PSCs (Figure 1), which may further potentiate the effect of stellate cells on chemoresistance.

## Hypoxia and chemoresistance

As mentioned above, hypoxia is a cancer driver that induces EMT, a phenotypic change associated with increased chemoresistance and invasiveness of cancer cells (Castellanos et al., 2013). In addition, hypoxia increases genomic instability by elevating intracellular levels of mutagenic reactive oxygen species and suppressing DNA repair (Bristow and Hill, 2008; Chan and Bristow, 2010). This microenvironment selects for cancer cell clones carrying mutations and phenotypic alterations that improve their survival, increase chemoresistance, and enhance migration out of the inhospitable microenvironment to metastatic sites (Bao et al., 2012).

The cellular effects of hypoxia are primarily initiated by hypoxia-induced factor-1 alpha (HIF-1α), a well-established marker for cells under hypoxic stress (Ke and Costa, 2006). HIF-1α contains an oxygen-responsive degradation domain that is hydroxylated under normoxic conditions by prolyl hydroxylases, leading to ubiquitination and rapid proteasomal degradation (Srinivas et al., 1999; Masson et al., 2001). Low oxygen levels inactivate these prolyl hydroxylases, stabilizing HIF-1α, allowing it to heterodimerize with HIF-1β, forming HIF-1, a transcription factor that regulates numerous genes responsible for the cellular response to hypoxia (Greijer et al., 2005; Semenza et al., 2006). HIF-1α has been detected in both pancreatic cancer cells and surrounding stromal cells, in pancreatic cancer tissue specimens (Shibaji et al., 2003; Ide et al., 2007; Sun et al., 2007; Hoffmann et al., 2008; Schwartz et al., 2010). Positive staining correlated with increased lymph node metastases, decreased apoptotic index, increased intratumoral microvessel density, advanced tumor stage and poorer overall survival (Shibaji et al., 2003; Ide et al., 2007; Sun et al., 2007; Hoffmann et al., 2008; Schwartz et al., 2010).

The influence of hypoxia on pancreatic cancer growth and metastatic spread has been more directly investigated in mouse models of the disease. Buchler et al. (2004) used an orthotopic mouse model of pancreatic cancer that develops distant metastases and measured primary tumor oxygenation using the Eppendorf histograph. The group found a significant correlation between lower tumor oxygenation and increased metastatic score (Buchler et al., 2004). Chang et al. (2011) later reiterated these results using orthotopic implants of pancreatectomy samples from pancreatic cancer patients. The group measured intratumoral hypoxia by immunohistochemistry using the hypoxia marker EF5, and observed a correlation of higher EF5 staining with rapid tumor growth, increased proliferation and increased metastases (Chang et al., 2011). Ide et al. (2007) suggested a potential molecular pathway for the increased invasiveness by associating increased levels of HIF1α with elevated paracrine signaling proteins hepatocyte growth factor and c-met in pancreatic cancer tissue specimens. Importantly, Salnikov et al. (2009) showed that while hypoxia drives EMT in both cancer stem cells and cancer cells, it is the stem cells that gain the more invasive phenotype.

Hypoxia in pancreatic cancer also selects for cancer cell clones with phenotypic changes that confer a survival advantage and consequently enhance their aggressiveness and chemoresistance. Erkan et al. (2005) nicely demonstrated this influence in their investigation of BNIP3, a hypoxia-inducible pro-apoptotic gene, in pancreatic cancer cells and pancreatic cancer patient samples. They firstly observed that downregulation of BNIP3 in patient samples correlated with poorer patient survival. Upon silencing BNIP3 in pancreatic cancer lines *in vitro*, the group observed a marked increase in chemoresistance to 5-FU and gemcitabine (Erkan et al., 2005). The results suggested that the hypoxic microenvironment imposes selective pressure that favors PC cells with the ability to bypass cell death mechanisms, and as a consequence, that can better resist chemotherapeutics. In addition, Arumugam et al. (2009) have demonstrated the link between hypoxia-induced EMT in pancreatic cancer cells and resistance to 5-FU, gemcitabine and cisplatin *in vitro*. Tumor cells under hypoxia are also driven to switch their metabolism from an oxygen-consuming pathway to a glycolytic pathway of ATP production (Chen et al., 2009). Such an environment selects for cells with enhanced glycolytic activity, as evidenced by increased levels of glycolytic enzymes in pancreatic cancer tissues and the correlation of glycolytic enzyme polymorphisms with poorer overall survival and enhanced tumor growth (Mikuriya et al., 2007; Dong et al., 2011).

PSCs are also influenced by hypoxia, leading to phenotypic changes that further stimulate pancreatic cancer cells and that facilitate the self-perpetuation of hypoxia. Masamune et al. (2008) investigated the effect of hypoxia on human CA-PSCs *in vitro*. When cultured under hypoxic conditions, CA-PSCs exhibited increased migration and type I collagen production, allowing perpetuation of fibrosis and hypoxia. Conditioned media from these cells also induced endothelial cell proliferation, migration and angiogenesis via increased PSC-mediated VEGF production, both *in vitro* and *in vivo* (Masamune et al., 2008). Spivak-Kroizman et al. (2013) showed that hypoxia also indirectly induces collagen secretion in PSCs, by increasing PC cell sonic hedgehog secretion. Erkan et al. (2009) reconciled the apparent contradictory role of PSCs in perpetuating hypoxia via induction of fibrosis, and in reducing it via pro-angiogenic signals. Using co-culture of PSCs and PC cells *in vitro*, the group showed that although hypoxia alone drives a proangiogenic PSC phenotype, activated PSCs induce PC cells to increase anti-angiogenic endostatin production, thus perpetuating hypoxia by inhibiting angiogenesis. More recently, Eguchi et al. (2013) demonstrated that hypoxia indirectly increases the invasiveness of PC cells *in vitro*, by inducing secretion of connective tissue growth factor from PSCs. It is therefore not surprising that many anti-angiogenic therapies result in increased metastases, as they create a hypoxic microenvironment that favors this phenotype (Ebos et al., 2009; Paez-Ribes et al., 2009). The major role of PSCs in establishing and perpetuating the hypoxic microenvironment makes them ideal therapeutic targets in pancreatic cancer. Given PSCs regulate ECM turnover, such an approach would ideally include co-administration of an anti-fibrotic reagent such as pirfenidone, to ensure fibrolysis occurs after targeting PSCs.

## Potential stromal targeting agents in the clinic

One reason drug trials have failed in pancreatic cancer is due to inclusion of all patients in a particular trial, regardless of the biological characteristics of an individual's tumor and no consideration of the stroma, which based on the evidence provided above is likely to influence a patient's response to chemotherapy. Understanding how stromal proteins influence drug resistance, drug delivery and patient survival has the potential to help clinicians make better use of available treatments to improve the outcome of patients with pancreatic cancer. Importantly, protein expression profiles of the stroma are now considered strong predictors of patient outcome (Conklin and Keely, 2012). For example, high stromal activity (αSMA positive PSCs) correlates with poor prognosis in pancreatic cancer patients (Erkan et al., 2008). Below we include discussion on a few of the promising stromal targeting therapies currently being investigated in the clinic.

At the 2013 ASCO meeting, a phase 1b study of gemcitabine plus PEGPH20 (PEGylated recombinant human hyularonidase) in patients with stage IV previously untreated pancreatic cancer was presented by Hingorani et al. (2013). PEGPH20 works by enzymatically depleting hyaluronic acid (a glycosaminoglycan), which is extremely abundant in pancreatic cancers and thought to contribute to the high interstitial fluid pressure (Thompson et al., 2010). The study separated out the nine patients with high levels of stromal hyaluronan and five of these patients showed a partial response to PEGPH20 treatment (response rate 56%). In addition, the PEGPH20 appeared to be well tolerated. The scientific and clinical community eagerly await the results of Phase II studies.

A Phase 3 trial is currently testing gemcitabine in combination with TH-302 in patients with locally advanced unresectable or metastatic pancreatic adenocarcinoma. As outlined above, pancreatic cancer is highly hypoxic and TH-302 is a chemotherapeutic agent which is selectively activated in a hypoxic tumor microenvironment (Sun et al., 2012).

Angiotensin II type I receptor inhibitors have also been explored as potential therapeutics to inhibit PSC activity. For example, Yamada et al. (2003) demonstrated that oral administration of candesartan (a widely used angiotensin II type-I receptor inhibitor) decreased ECM production and αSMA expression (activated PSC marker) in a rat chronic pancreatitis model. Furthermore, a retrospective clinical study suggested that pancreatic cancer patients treated with angiotensin II type 1 receptor inhibitors in combination with gemcitabine may have improved clinical outcome (Nakai et al., 2010). To expand on these findings the same authors recently completed a multicenter phase II clinical trial (35 patients with advanced pancreatic cancer) to examine whether patients treated with candesartan in combination with gemcitabine would have improved survival. The treatment regime was tolerated using moderate doses of candesartan with gemcitabine, but failed to demonstrate any significant clinical activity (Nakai et al., 2013).

More recently, in an elegant and comprehensive study by Chauhan et al. (2013), the angiotensin II Type 1 receptor inhibitor losartan was found to reduce stromal collagen and hyaluronan production, as well as decrease the number of αSMA positive PSCs in orthotopic pancreatic tumors. As a result, the treatment of tumors with losartan reduced GISS and increased vascular perfusion. The overall outcome was reduced hypoxia and increased sensitivity to chemotherapy agents. This promising pre-clinical study has now moved forward to a clinical trial in pancreatic cancer (NCT01821729). Notably, losartan has been shown to have a higher tumor penetration when compared to other angiotensin II Type 1 receptor inhibitors including candesartan. Indeed, inadequate tumor penetration of candesartan may in part explain its modest effect on chemosensitivity in the recent clinical trial (Nakai et al., 2013).

A recent article by Kozono et al. (2013), demonstrated that an anti-fibrotic agent pirfenidone decreased tumor growth/metastases and increased drug sensitivity in a mouse orthotopic model of pancreatic cancer (co-injection of PSCs and tumor cells into the pancreas). Pirfenidone is currently approved for the treatment of idiopathic pulmonary fibrosis in Europe and based on evidence by Kozono et al. (2013), it may be a promising anti-fibrotic agent which can be repurposed to reduce the activity of PSCs in pancreatic cancer.

Finally, Nab-paclitaxel or Abraxane^®^ (Abraxis Bioscience) has generated great interest as a novel therapeutic for pancreatic cancer. A recent phase III MPACT (Metastatic Pancreatic Adenocarcinoma Clinical Trial) trial demonstrated that the addition of Nab-paclitaxel with gemcitabine was able to significantly improve the median survival of metastatic pancreatic cancer patients (8.5 months) when compared to gemcitabine treated only arm (6.7 months) and reduce toxicities (neuropathy and neutropenia) commonly associated with the cremaphor formulation used to dissolve paclitaxel thereby, allowing for a higher paclitaxel dose to be delivered to the patient (Von Hoff et al., 2013). Interestingly, several studies have also shown that Nab-paclitaxel alone or in combination with gemcitabine depletes PCSs and desmoplastic stroma (Von Hoff et al., 2011; Alvarez et al., 2013). Moreover, it is hypothesized that the albumin-bound Nab-paclitaxel may selectively accumulate in the pancreatic stroma via its binding to secreted protein acidic and rich in cysteine (SPARC) matricellular glycoprotein which binds albumin and is overexpressed in tumor stroma. Indeed, high SPARC expression has been correlated to poor survival outcome and has been suggested as a possible predictive biomarker for Nab-paclitaxel (Von Hoff et al., 2011; Alvarez et al., 2013). However, a recent study by Neesse et al. (2013) showed that the effects of Nab-paclitaxel were largely dose-dependent and that SPARC expression in the tumor stroma did not influence drug accumulation in a pancreatic cancer mouse model. However, they did report increased Nab-paclitaxel concentrations in plasma suggesting a potential interaction with circulating SPARC. Therefore, it is possible that high circulating SPARC may aid in increased drug retention and tissue delivery. Future studies will be needed to evaluate tissue and plasma SPARC expression as a predictive biomarker for Nab-paclitaxel.

## Conclusions

**Figure 3:**
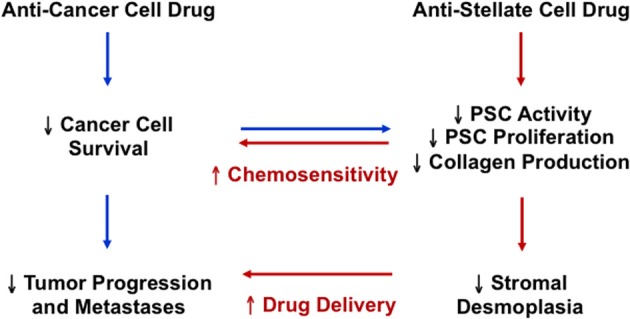
**The potential therapeutic implication of targeting both pancreatic cancer cells and pancreatic stellate cells in pancreatic cancer**.

As outlined above, a significant body of work corroborates the notion that CA-hPSCs are not relegated to a bystander role in PDAC chemoresistance, but instead, they are critical in driving it via both direct and indirect mechanisms. This occurs by way of PSC generated fibrosis creating a hypoxic microenvironment that directly fuels the chemoresistance of cancer cells. Concurrently, fibrosis impairs effective drug perfusion to cancer cells, which indirectly augments chemoresistance. Furthermore, there is mounting evidence that not only is stromal depletion therapeutically possible in mice with pancreatic tumors, but it also facilitates the delivery of chemotherapeutics to pancreatic tumors. Notably, stromal depletion entails a reversion of fibrosis as well as a decline in the number of cells generating fibrosis because reducing the number of activated PSCs in turn would diminish collagen production. Thus, in light of their major contribution to fibrosis in pancreatic cancer as well as their highly proliferative capacity, cancer-associated PSCs are ideal candidates for the identification of stromal-related therapeutic targets that may act as additional targets for potentiating existing chemotherapeutics (Figure 3).

### Conflict of interest statement

The authors declare that the research was conducted in the absence of any commercial or financial relationships that could be construed as a potential conflict of interest.

## Abbreviations

CA-hPSC: cancer-associated human pancreatic stellate cell
COX-2: cyclooxygenase-2
CTGF: connective tissue growth factor
PSC: pancreatic stellate cell
ECM: extracellular matrix
EMMPRIN: extracellular matrix metalloproteinase inducer
EMT: epithelial-mesenchymal transition
ET-1: endothelin 1
IFP: interstitial fluid pressure
IL: interleukin
PDAC: pancreatic ductal adenocarcinoma
GISS: growth-induced solid stress
MMPs: matrix metalloproteinases
PDGF: platelet-derived growth factor
TGFβ: transforming growth factor β
TNFα: tumor necrosis factor α
TRAIL: TNF-related apoptosis-inducing ligand HIF-1α, hypoxia-induced factor-1 alpha.
